# Assessment of Potential Drug‐Drug Interactions in Hospitalized Patients With Cardiovascular Diseases at Kenyatta National Hospital, Nairobi, Kenya: A Cross‐Sectional Study

**DOI:** 10.1002/hsr2.73356

**Published:** 2026-10-08

**Authors:** Brenda Koech, Ermias Terefe

**Affiliations:** ^1^ Department of Pharmacology, Pharmacognosy and Pharmaceutical Chemistry, School of Pharmacy and Health Sciences United States International University‐Africa Nairobi Kenya

**Keywords:** cardiovascular diseases, drug–drug interactions, Kenyatta National Hospital, medication safety, pharmacovigilance, polypharmacy

## Abstract

**Background and Aim:**

Drug–drug interactions (DDIs) occur when the pharmacological effect of one medication is altered by the concurrent administration of another, potentially leading to reduced therapeutic efficacy or increased risk of adverse effects. Hospitalized patients with cardiovascular diseases are particularly vulnerable due to complex treatment regimens and polypharmacy. This study aimed to assess the prevalence, severity, and management of potential drug–drug interactions (pDDIs) among cardiovascular inpatients at Kenyatta National Hospital (KNH), Kenya.

**Methods:**

This retrospective cross‐sectional study included 100 medical records of adult cardiovascular inpatients (hypertension, cerebrovascular accident, coronary artery disease, heart failure) at Kenyatta National Hospital (September 2022–April 2023), selected using systematic sampling. Socio‐demographic, comorbidity, and medication data were extracted using a structured abstraction tool. Potential drug–drug interactions were identified using Lexicomp® and Medscape®. Data were analyzed in SPSS version 20, using descriptive statistics and chi‐square tests to assess associations between patient characteristics and the occurrence of pDDIs.

**Results:**

Among 100 hospitalized cardiovascular patients (56% male, 44% female; mean age 62.5 ± 10.2 years), a high prevalence of pDDIs was observed, with 91% of patients experiencing at least one interaction (95% CI: 83.8–95.2%). Among affected patients, the majority were classified as moderate severity based on each patient's single most severe interaction (62.6%), followed by minor (24.2%) and severe (13.2%). Hypertension was the most common comorbidity. An exact Freeman‐Halton test showed a statistically significant association between the number of prescribed medications and the occurrence of pDDIs (*p* < 0.001), with a Cochran‐Armitage trend test confirming a dose‐response relationship (Z = 3.54, *p* < 0.001), whereas no significant association was observed with age or sex. Commonly prescribed cardiovascular drug combinations, particularly involving antiplatelets, anticoagulants, and antihypertensive agents, were frequently associated with clinically relevant interactions.

**Conclusion:**

Potential drug–drug interactions are highly prevalent among hospitalized cardiovascular patients at Kenyatta National Hospital, with most interactions requiring monitoring or therapeutic adjustment. Strengthening routine medication review, integrating drug interaction screening tools, and promoting interdisciplinary collaboration may enhance medication safety and optimize patient care in this population.

## 1 Introduction

Cardiovascular diseases (CVDs) remain the leading cause of morbidity and mortality worldwide, accounting for approximately 19.8 million deaths annually and nearly 32% of all global deaths [1]. Among these, myocardial infarction and stroke contribute to the majority of fatalities, highlighting the substantial global burden of cardiovascular conditions [2]. The increasing prevalence of non‐communicable diseases has led to more complex therapeutic regimens, particularly in hospitalized patients, where multiple medications are often prescribed concurrently to manage comorbidities and prevent disease progression.

Polypharmacy, commonly defined as the concurrent use of multiple medications, is highly prevalent among cardiovascular patients and significantly increases the risk of drug–drug interactions (DDIs) [3, 4]. DDIs occur when one drug alters the pharmacokinetic or pharmacodynamic properties of another, potentially leading to reduced therapeutic efficacy or increased toxicity [5]. In clinical practice, particularly among patients with cardiovascular diseases who are frequently prescribed combinations of antihypertensives, anticoagulants, antiplatelets, and lipid‐lowering agents, DDIs represent a major challenge in medication management [6]. Failure to recognize and appropriately manage these interactions may result in adverse clinical outcomes, including treatment failure, increased toxicity, prolonged hospitalization, and higher healthcare costs [7, 8, 9]

Adverse drug events (ADEs), many of which are associated with DDIs, remain a significant cause of hospital admissions and medication‐related morbidity, particularly in low‐ and middle‐income countries where pharmacovigilance systems may be limited [10]. Identifying and managing potential drug–drug interactions (pDDIs) is therefore essential for improving patient safety and optimizing therapeutic outcomes. Previous studies have reported a high prevalence of pDDIs among hospitalized cardiovascular patients, with estimates ranging from 60% to over 90%, depending on the clinical setting and detection tools used [11, 12, 13]). For example, a cross‐sectional study conducted in a cardiothoracic intensive care unit in China reported that 84.2% of patients experienced at least one DDI, with an average of 3.4 interactions per patient [14].

Several risk factors have been consistently associated with the occurrence of DDIs in cardiovascular patients, including advanced age, presence of multiple comorbidities such as diabetes and hypertension, and the use of multiple medications (polypharmacy) [5]. Additionally, variability in drug metabolism, particularly due to genetic differences in cytochrome P450 enzyme activity, further complicates the prediction and clinical impact of DDIs [15]. These interactions may lead to serious clinical consequences such as bleeding, hypotension, arrhythmias, renal impairment, and reduced effectiveness of essential cardiovascular therapies [16]. As a result, continuous monitoring and active involvement of clinical pharmacists are essential to identify, prevent, and manage DDIs in hospital settings.

Despite the growing body of international evidence, there remains limited data on the prevalence, patterns, and clinical implications of drug–drug interactions among hospitalized cardiovascular patients in Kenya. Understanding the distribution of pDDIs across patient demographics, clinical characteristics, and medication profiles is crucial for informing clinical decision‐making and strengthening medication safety practices in resource‐limited settings.

Therefore, this study aimed to assess the prevalence, severity, and management of potential drug–drug interactions among hospitalized patients with cardiovascular diseases at Kenyatta National Hospital (KNH), Nairobi, Kenya. In addition, the study explored the distribution of pDDIs across patient characteristics and medication patterns to enhance the clinical relevance of the findings and support the development of strategies for safer prescribing practices.

## 2 Methods

### 2.1 Study Design

This study employed a retrospective cross‐sectional design to evaluate the prevalence and patterns of potential drug–drug interactions (pDDIs) among hospitalized patients with cardiovascular diseases at Kenyatta National Hospital (KNH). Eligible patient medical records were selected using a systematic sampling approach. Extracted medication data were analyzed using drug interaction screening tools to identify and classify potential interactions.

Potential drug–drug interactions were identified using Lexicomp® and Medscape® drug interaction databases. Lexicomp® was used as the primary reference due to its established reliability and widespread use in clinical pharmacology practice, while Medscape® was used as a supplementary tool to cross‐check interaction severity and enhance classification consistency. The use of multiple drug interaction databases has been recommended in pharmacovigilance research to improve the accuracy and reliability of DDI identification [17].

### 2.2 Study Setting and Period

The study was conducted at Kenyatta National Hospital (KNH), Nairobi, Kenya, the largest national referral and teaching hospital in the country, with an approximate bed capacity of 2,400 and an annual patient volume of 949,000 inpatients and 800,000 outpatients, as officially reported by the hospital [18]. The hospital serves a diverse patient population from across Kenya and neighboring East and Central African countries, making it an appropriate setting for studying medication‐related safety issues in routine clinical practice.

Medical records of patients admitted between September 2022 and April 2023 were reviewed. This setting provided a suitable environment for assessing the prevalence and patterns of potential drug–drug interactions among hospitalized cardiovascular patients.

### 2.3 Target Population

The target population comprised medical records of adult patients diagnosed with cardiovascular diseases and admitted to Kenyatta National Hospital during the study period. Cardiovascular conditions included hypertension, cerebrovascular accidents (CVA), coronary artery disease, heart failure, cardiomyopathies, and other related cardiovascular disorders as documented in patient records.

### 2.4 Eligibility Criteria

Patients were eligible for inclusion if they were aged 18 years or older, had a confirmed diagnosis of cardiovascular disease, and were prescribed at least two concurrent medications during hospitalization. Only records with complete and detailed clinical information, including medication history and treatment data, were included in the analysis.

Patients were excluded if they were younger than 18 years or had incomplete records lacking essential information required for the assessment of potential drug–drug interactions. These criteria were applied to ensure data completeness, accuracy, and reliability of the study findings.

### 2.5 Sampling and Sample Size Determination

Eligible treatment sheets from the Kenyatta National Hospital Health Information Department covering the study period (September 2022 to April 2023) were arranged chronologically according to the date of admission. A systematic sampling approach was employed whereby every second eligible treatment sheet was selected, beginning with the first eligible record in the chronological sequence, until the target sample of 100 treatment sheets was obtained. Treatment sheets were screened against the predefined inclusion and exclusion criteria before data extraction.

The sample size was initially calculated using the single population proportion formula:

where *n* is the required sample size, *Z* is the standard normal value corresponding to a 95% confidence level (1.96), *P* is the estimated prevalence (assumed to be 50% due to the absence of prior local data), and *d* is the margin of error (5%). Based on these assumptions, the calculated sample size was 384 medical records.

Although an initial sample size calculation indicated that a larger sample would provide greater statistical precision, the study was conducted using a sample of 100 treatment sheets because of the practical resource and time constraints associated with this single‐center undergraduate research project. The smaller sample size may have reduced the precision and generalization of the findings, and this limitation is acknowledged in the discussion.

### 2.6 Study Variables

The primary outcome variable in this study was the occurrence of potential drug–drug interactions (pDDIs) among hospitalized cardiovascular patients. The severity of identified pDDIs, categorized as severe, moderate, or minor, was also assessed.

Independent variables included socio‐demographic characteristics such as age, sex, and residential area, as well as clinical factors including the presence of comorbidities and the number of prescribed medications. These variables were analyzed to explore their relationship with the occurrence and severity of pDDIs in the study population. Sex was recorded as documented in the patient's clinical file (male/female as noted by the attending clinician at admission) and was not self‐reported by the patient for the purposes of this study.

### 2.7 Data Collection Methods

A structured data abstraction form was developed based on previously published studies on drug–drug interactions and clinical pharmacology research. The form was designed to systematically capture relevant information from patient medical records, including socio‐demographic characteristics, prescribed medications, and concomitant diseases. To ensure content validity, the data abstraction form was reviewed by senior pharmacology experts prior to data collection.

Potential drug–drug interactions (pDDIs) were identified by screening prescribed medications using Lexicomp® and Medscape® drug interaction databases. Lexicomp® was used as the primary reference for identifying and classifying the severity of potential drug‐drug interactions because of its widespread use and established reliability in clinical practice. Medscape® was consulted as a supplementary reference to confirm the presence of identified interactions. Where discrepancies in severity classification occurred between the two databases, the Lexicomp® classification was retained as the primary classification for analysis. The use of multiple drug interaction databases has been recommended to improve the accuracy and reliability of DDI identification in clinical research [19, 20, 21].

### 2.8 Pretesting of Data Collection Tool

To ensure the reliability and practicality of the data abstraction process, the data collection tool was pretested on 5% of the total sample size prior to the main data collection phase. The pretesting process enabled the assessment of clarity, completeness, and consistency of the tool in capturing relevant variables from patient medical records. It also helped identify potential ambiguities and inconsistencies in data extraction. Based on the findings from the pilot phase, necessary modifications were made to improve the structure, clarity, and usability of the abstraction form.

### 2.9 Statistical Analysis

Data were entered and analyzed using SPSS version 20.0. Descriptive statistics, including frequencies, percentages, means, and standard deviations, were used to summarize socio‐demographic characteristics, clinical conditions, medication patterns, prevalence of potential drug–drug interactions (pDDIs), and their severity categories.

Chi‐square tests or Fisher's exact tests, where appropriate, were used to assess associations between categorical variables, including age group, sex, number of prescribed medications, and the occurrence of pDDIs. Fisher's exact test was used in place of the chi‐square test whenever an expected cell count was below 5, and the Freeman‐Halton extension of Fisher's exact test was used for the corresponding comparison involving a contingency table larger than 2 × 2. A Cochran‐Armitage trend test was additionally performed to assess a dose‐response relationship across the ordinal medication‐count categories. Effect size was assessed using Cramér's V. Statistical significance was set at *p* < 0.05 (two‐sided).

This study is reported with reference to the STROBE guideline for cross‐sectional studies; a completed STROBE checklist mapping each item to the corresponding manuscript section is provided as a supplementary file.

### 2.10 Ethical Considerations

Ethical approval for the study was obtained from the United States International University‐Africa Institutional Review Board (USIU‐A/IRB/SS03/2023), the Kenyatta National Hospital Ethics and Research Committee, and the National Commission for Science, Technology and Innovation (NACOSTI; Permit No. NACOSTI/P/23/23349).

Permission to access patient medical records was granted by the Health Information Department at Kenyatta National Hospital. Patient confidentiality was strictly maintained by de‐identifying all personal information, and only anonymized data were used for analysis.

The study was conducted in accordance with established ethical standards for medical research involving human subjects, ensuring the protection of participant privacy and data confidentiality.

## 3 Results

### 3.1 Socio‐Demographic and Clinical Characteristics of Patients

A total of 100 medical records of hospitalized patients with cardiovascular diseases were included in the study. Of these, 56% were male and 44% were female. The mean age of the patients was 62.5 ± 10.2 years, with an age range of 18 to 85 years. The majority of patients (65%) were aged above 50 years, indicating that cardiovascular conditions were more common among older individuals in this study population.

Regarding socio‐demographic characteristics, most participants were married (45%), followed by widowed (21%), single (10%), separated (5%), and divorced (4%). Marital status was not documented for 15% of patients. In terms of residence, 53% of patients were from Nairobi, while 47% were from areas outside Nairobi (Table 1).

**Table 1:** Socio‐demographic Characteristics of Study Participants (*n* = 100).

Variable	Category	Frequency (*n*)	Percentage (%)
Age (years)	18–30	4	4.0
	31–50	31	31.0
	> 50	65	65.0
Marital status	Married	45	45.0
	Single	10	10.0
	Widowed	21	21.0
	Separated	5	5.0
	Divorced	4	4.0
	Not documented	15	15.0
Residential area	Nairobi	53	53.0
	Outside Nairobi	47	47.0

*Note:* Percentages are based on the total study population (*n* = 100).

Comorbid conditions were highly prevalent among the study participants. Hypertension was the most common condition, affecting 78% of patients, followed by cerebrovascular accidents (CVA) in 56% and diabetes mellitus in 19% of patients. Other cardiovascular‐related conditions included coronary heart disease, dilated cardiomyopathy, and hypertensive heart disease. The presence of multiple comorbidities reflects the complexity of patient management in this population (Table 2).

**Table 2:** Frequency of comorbid conditions among hospitalized cardiovascular patients at Kenyatta National Hospital (*n* = 100).

Comorbidity	Frequency (*n*)	Percentage (%)^a^
Hypertension	78	78.0
Cerebrovascular accident (CVA)	56	56.0
Diabetes mellitus (DM)	19	19.0
Dilated cardiomyopathy	12	12.0
Acute kidney injury (AKI)	10	10.0
Renal vascular disease (RVD)	9	9.0
Pneumonia	8	8.0
Epilepsy	8	8.0
HIV infection	7	7.0
Coronary heart disease	4	4.0
Hyperthyroidism	4	4.0
Deep vein thrombosis (DVT)	2	2.0
Chronic lung disease (e.g., fibrosis, cor pulmonale)	2	2.0
Sepsis	1	1.0
Diabetic ketoacidosis (DKA)	1	1.0
Others (e.g., BPH, GI bleeding, dementia)	5	5.0

Abbreviations: AKI, acute kidney injury; BPH, benign prostatic hyperplasia; CVA, cerebrovascular accident; DKA, diabetic ketoacidosis; DM, diabetes mellitus; DVT, deep vein thrombosis; GI, gastrointestinal; RVD, renal vascular disease.

^a^ Patients could have more than one comorbid condition; therefore, percentages exceed 100%.

### 3.2 Prevalence of Potential Drug–Drug Interactions

A high prevalence of potential drug–drug interactions (pDDIs) was observed among the study population. Out of the 100 hospitalized cardiovascular patients included in the study, 91% had at least one identified potential drug–drug interaction (95% CI: 83.8–95.2%).

A total of 406 severe‐ or moderate‐severity potential drug–drug interactions were individually documented by drug combination (Supplementary Table S1), corresponding to a mean of 4.46 severe/moderate interactions per patient among those with at least one identified pDDI (*n* = 91). An additional minimum of 22 minor‐severity interactions were identified in patients whose most severe interaction was classified as minor (Table 4), giving a conservative overall minimum of 428 total interactions (mean ≥ 4.70 per affected patient). As the original per‐patient interaction log was not retained for the present analysis, an exact total interaction count and median could not be calculated; this is noted as a limitation.

These findings indicate that exposure to potential drug–drug interactions was highly common among hospitalized cardiovascular patients in this setting, with several patients experiencing multiple interactions.

The prevalence of pDDIs among study participants is summarized in Table 3, while the severity classification of identified pDDIs is presented in Table 4.

**Table 3:** Prevalence of potential drug–drug interactions among hospitalized cardiovascular patients at Kenyatta National Hospital (*n* = 100).

pDDI status	Frequency (*n*)	Percentage (%)
Patients with at least one pDDI	91	91.0
Patients without pDDI	9	9.0
Total	100	100.0

*Note:* pDDIs were identified using Lexicomp® and Medscape® drug interaction databases.

Abbrevation: pDDI, potential drug–drug interaction.

**Table 4:** Severity classification of identified potential drug–drug interactions among hospitalized cardiovascular patients (*n* = 91).

Severity category	Frequency (*n*)	Percentage (%)
Severe	12	13.2
Moderate	57	62.6
Minor	22	24.2
Total	91	100.0

*Note:* Severity classification was based on Lexicomp® and Medscape® drug interaction databases.

### 3.3 Classification and Most Frequently Identified Potential Drug–Drug Interactions

The identified potential drug–drug interactions (pDDIs) were classified according to their clinical severity. Among the interactions detected, 13.2% were classified as severe (95% CI: 7.7–21.6%), 62.6% as moderate (95% CI: 52.4–71.9%), and 24.2% as minor (95% CI: 16.5–33.9%). Moderate interactions constituted the largest proportion of pDDIs observed in this study.

Each patient was classified according to the single most severe potential drug–drug interaction identified in their medication profile (Table 4). This is distinct from the itemized interaction‐level tally presented in Supplementary Table S1, which records the frequency of each specific drug combination independent of patient‐level severity classification.

This severity distribution indicates that a substantial proportion of interactions required clinical attention, including close monitoring or therapeutic adjustment. Severe interactions, although less frequent, represent clinically important combinations that may pose significant risks if not appropriately managed.

The most commonly observed interacting drug combinations and their corresponding severity classifications are presented in Table 5.

**Table 5:** Most frequently identified potential drug–drug interactions and recommended management strategies among hospitalized cardiovascular patients.

Drug combination	Mechanism	Severity	Recommended action	Frequency, *n* (%)^a^
Aspirin + Clopidogrel	Additive antiplatelet effect resulting in increased bleeding risk	Moderate	Monitor closely	29 (7.1)
Enoxaparin + Clopidogrel	Additive anticoagulant effect resulting in increased bleeding risk	Moderate	Monitor closely	25 (6.2)
Enalapril + Spironolactone	Increased risk of hyperkalemia	Moderate	Monitor serum potassium levels	15 (3.7)
Enalapril + Furosemide	Increased risk of hypotension and renal impairment	Moderate	Monitor closely	20 (4.9)
Diclofenac + Enalapril	Reduced antihypertensive effect of enalapril	Severe	Avoid combination or use an alternative agent	18 (4.4)
Esomeprazole + Clopidogrel	Cytochrome P450 2C19 inhibition resulting in reduced clopidogrel activation	Severe	Avoid combination or use an alternative agent	22 (5.4)
Levofloxacin + Ondansetron	Increased risk of QT interval prolongation	Severe	Avoid combination or use an alternative agent	14 (3.4)
Phenytoin + Nifedipine	Cytochrome P450 3A4 induction resulting in reduced nifedipine exposure	Severe	Avoid combination or use an alternative agent	12 (3.0)
Nifedipine + Amlodipine	Additive vasodilatory effects and potential pharmacokinetic interaction	Severe	Avoid or use alternative	28 (6.9)
Aspirin + Warfarin	Increased anticoagulant effect and bleeding risk	Moderate	Monitor closely	20 (4.9)

*Note:* Severity classification and recommended management actions were based on Lexicomp® and Medscape® drug interaction databases.

^a^ The frequency (n, %) column reflects the number of times each combination was documented in Supplementary Table S1, expressed as a percentage of the 406 total severe‐ or moderate‐severity potential drug–drug interactions identified in this study (Section 3.2).

### 3.4 Management of Identified Potential Drug–Drug Interactions

Based on database‐derived recommendations from the identified potential drug–drug interactions (pDDIs), several management strategies were suggested. Among the 25 individual drug combinations documented in Supplementary Table S1, 8 severe combinations (121 occurrences) each carried a recommendation to avoid the combination or substitute an alternative agent, 16 moderate combinations (280 occurrences) each carried a recommendation for close clinical or laboratory monitoring, and 1 combination (Efavirenz + Carvedilol, 5 occurrences) was classified as a potential weak interaction for which clinical monitoring was recommended without an a priori dosage adjustment; none of the identified combinations carried a specific dosage‐adjustment recommendation.

These management recommendations were derived from the complete set of identified pDDIs and reflect the range of clinical actions required to mitigate potential interaction‐related risks. It is important to note that some interactions may require more than one management approach.

A comprehensive list of all identified drug–drug interactions and their corresponding management recommendations is provided in Supplementary Table S1, extending beyond the most frequently observed interactions presented in Table 5.

Overall, these findings highlight the need for active medication review and appropriate clinical decision‐making when managing potential drug–drug interactions in hospitalized cardiovascular patients.

### 3.5 Association Between Patient Characteristics, Medication Burden, and pDDIs

Stratified descriptive analysis was conducted to examine the distribution of potential drug–drug interactions (pDDIs) across selected patient characteristics. The findings showed that pDDIs were more frequently observed among older patients, with the highest proportion occurring in those aged above 50 years; however, this difference was not statistically significant. Additionally, pDDIs were observed in both male and female patients, with comparable proportions across the two groups. This distribution across age and sex is presented in Table 6.

**Table 6:** Association between selected patient characteristics and occurrence of potential drug–drug interactions among hospitalized cardiovascular patients.

Variable	Category	Total (*n*)	pDDI present, *n* (%)	*χ* ^2^	df	*p*‐value	Cramér's V
Age group	≤ 50 years	35	30 (85.7)	1.89	1	0.169	0.14
	> 50 years	65	61 (93.8)	—	—	—	—
Sex	Male	56	52 (92.9)	0.51	1	0.476	0.07
	Female	44	39 (88.6)	—	—	—	—

Abbrevations: Cramér's V, measure of association effect size; df, degrees of freedom; pDDI, potential drug–drug interaction; χ^2^, chi‐square statistic.

Furthermore, the severity of pDDIs varied across patient groups, with moderate interactions being the most frequently observed category across all subgroups. A relatively higher proportion of severe interactions was observed among patients with higher medication burden and multiple comorbidities. The distribution of pDDI severity across patient characteristics is presented in Table 7.

**Table 7:** Distribution of potential drug–drug interaction severity according to patient characteristics among hospitalized cardiovascular patients.

Variable	Category	Severe (%)	Moderate (%)	Minor (%)
Age group	≤ 50 years (*n* = 35)	10	60	30
	> 50 years (*n* = 65)	15	65	20
Sex	Male (*n* = 56)	14	63	23
	Female (*n* = 44)	12	61	27

*Note:* Percentages represent the distribution of pDDI severity within each patient subgroup.

Chi‐square tests were performed to examine associations between patient characteristics and pDDI occurrence. No statistically significant association was observed between age group (≤ 50 vs > 50 years) and pDDI occurrence (*χ* ^2^(1) = 1.89, *p* = 0.169), or between sex and pDDI occurrence (*χ* ^2^(1) = 0.51, *p*= 0.476). The 50‐year threshold was selected a priori to distinguish middle‐aged from older adult patients, consistent with age‐stratification cut‐offs commonly applied in cardiovascular pharmacoepidemiology research in similar settings. A statistically significant association was identified between the number of prescribed medications and pDDI occurrence. Because the ≥ 8‐medications stratum contained an expected cell count below 5, the chi‐square approximation was not valid for this comparison; an exact Freeman‐Halton test was therefore applied (*p *< 0.001). A Cochran‐Armitage trend test across the three ordinal medication‐count categories was also significant (Z = 3.54, *p* < 0.001), supporting a dose‐response relationship between the number of prescribed medications and pDDI occurrence. These findings are presented in Table 6 and Table 8. Overall, these findings highlight important patterns in the occurrence and severity of pDDIs across different patient groups.

**Table 8:** Association between number of prescribed medications and occurrence of potential drug–drug interactions among hospitalized cardiovascular patients.

Number of medications	Patients (*n*)	pDDI present, *n* (%)	*χ* ^2^	df	*p*‐value	Cramér's V
2–4 drugs	20	14 (70.0)	N/A	N/A	*p* < 0.001 (Freeman‐Halton)	N/A
5–7 drugs	45	42 (93.3)				
≥ 8 drugs	35	35 (100.0)				

Abbrevations: Cramér's V, measure of association effect size; df, degrees of freedom; pDDI, potential drug–drug interaction; χ^2^, chi‐square statistic.

## 4 Discussion

### 4.1 Overview of Key Findings

This study demonstrated a high prevalence of potential drug–drug interactions (pDDIs) among hospitalized patients with cardiovascular diseases, with 91% of patients experiencing at least one interaction. The majority of identified pDDIs were of moderate severity, indicating that while many interactions may not result in immediate clinical harm, they require careful monitoring and potential therapeutic adjustments. Several clinically important drug combinations involving antiplatelet agents, anticoagulants, and antihypertensive medications were frequently observed.

Stratified analysis showed variations in the occurrence of pDDIs across patient subgroups. Although a higher proportion of pDDIs was observed among older patients, this association was not statistically significant. In contrast, a statistically significant association was identified between the number of prescribed medications and the occurrence of pDDIs, highlighting polypharmacy as a key factor associated with increased risk of drug–drug interactions in this population.

These findings underscore the importance of careful medication review and monitoring, particularly among patients receiving multiple medications, to minimize the risk of clinically relevant drug–drug interactions.

The identification of a potential drug–drug interaction does not necessarily imply inappropriate prescribing. In cardiovascular practice, several of the combinations identified in this study, particularly dual antiplatelet therapy and concomitant antiplatelet–anticoagulant regimens, as well as the concurrent use of low‐dose aspirin with an ACE inhibitor in patients with coronary artery disease or heart failure, are recommended by evidence‐based clinical guidelines for selected indications despite their known interaction potential. Accordingly, the prevalence reported in this study should be interpreted as reflecting exposure to potential drug–drug interactions requiring appropriate clinical monitoring and risk mitigation, rather than as a direct measure of prescribing quality or medication error.

### 4.2 Prevalence of Potential Drug–Drug Interactions

The high prevalence of potential drug–drug interactions (pDDIs) observed in this study is consistent with findings from previous studies conducted in both high‐income and low‐ and middle‐income settings. Reported prevalence rates among hospitalized cardiovascular patients typically range from 60% to over 90%, reflecting the substantial burden of medication‐related risks in this population [22, 23, 24].

The elevated prevalence identified in this study may be attributed to the complexity of cardiovascular disease management, which often requires the concurrent use of multiple drug classes, including antihypertensives, antiplatelets, anticoagulants, and lipid‐lowering agents. These therapeutic combinations increase the likelihood of both pharmacokinetic and pharmacodynamic interactions, particularly among patients with multiple comorbidities and advanced age [24, 25].

Additionally, the high prevalence of pDDIs may reflect gaps in routine medication review practices and limited integration of clinical decision support systems in some healthcare settings. Similar observations have been reported in recent pharmacovigilance studies, which emphasize the importance of systematic DDI screening and monitoring to enhance medication safety in hospital environments [5, 12].

### 4.3 Severity and Clinical Significance of Potential Drug–Drug Interactions

The predominance of moderate‐severity classifications (62.6%) among affected patients, based on each patient's single most severe interaction, observed in this study suggests that while many potential drug–drug interactions (pDDIs) may not result in immediate clinical harm, they require careful monitoring and, in some cases, therapeutic adjustment to prevent adverse outcomes. Moderate interactions often involve pharmacodynamic or pharmacokinetic mechanisms that can gradually compromise treatment efficacy or safety if left unaddressed. In contrast, severe interactions, although less frequent (13.2%), represent clinically important combinations that may pose significant risks if not appropriately managed.

These findings are consistent with previous studies, which have similarly reported a higher proportion of moderate interactions compared to severe interactions in hospitalized cardiovascular populations [26, 27, 28]). The predominance of moderate interactions across studies may reflect the widespread use of combination therapies that require monitoring rather than immediate discontinuation.

Variations in the distribution of severity categories between studies may be attributed to differences in prescribing practices, patient demographics, disease burden, and the drug interaction screening tools employed [17, 29, 30].

From a clinical perspective, the high proportion of moderate and severe pDDIs underscores the importance of routine medication review, particularly in high‐risk populations such as elderly patients and those with multiple comorbidities. Early identification and appropriate management of these interactions are essential to minimize potential risks and optimize therapeutic outcomes.

### 4.4 Common Drug–Drug Interactions and Clinical Implications

The most frequently identified drug combinations in this study, as presented in Table 5, involved antiplatelet agents (e.g., aspirin and clopidogrel), anticoagulants, antihypertensive agents, and nonsteroidal anti‐inflammatory drugs (NSAIDs). These drug classes are routinely co‐prescribed in the management of cardiovascular diseases; however, their concurrent use substantially increases the risk of clinically significant drug–drug interactions.

In particular, combinations such as aspirin and clopidogrel, as well as anticoagulants used alongside antiplatelet agents, are associated with an increased risk of bleeding due to additive pharmacodynamic effects on platelet aggregation and coagulation pathways. Similarly, interactions between antihypertensive agents and NSAIDs may reduce therapeutic efficacy through inhibition of prostaglandin‐mediated vasodilation, potentially leading to blood pressure destabilization and renal impairment. Furthermore, combinations involving beta‐blockers and calcium channel blockers may produce additive cardiovascular effects, including hypotension and bradycardia, due to their synergistic action on cardiac conduction and contractility.

These findings are consistent with previous studies that have identified similar drug classes as major contributors to clinically significant pDDIs in cardiovascular patients [17, 31, 32]. The recurrent involvement of these high‐risk drug combinations underscores the importance of careful prescribing practices and the need for ongoing medication review in patients receiving complex cardiovascular therapies.

From a clinical perspective, increased awareness of these commonly occurring high‐risk interactions is essential for healthcare providers to implement appropriate risk mitigation strategies. These may include dose optimization, substitution with safer therapeutic alternatives, and enhanced clinical and laboratory monitoring to minimize potential adverse outcomes.

### 4.5 Distribution of Potential Drug–Drug Interactions Across Patient Characteristics

The stratified analysis conducted in this study revealed important patterns in the distribution of potential drug–drug interactions (pDDIs) across patient characteristics. Although a higher proportion of pDDIs was observed among older patients, this association was not statistically significant. This trend is consistent with existing literature suggesting that age‐related physiological changes, including alterations in drug metabolism and excretion, may increase susceptibility to drug interactions [33, 34, 35].

In contrast, patients receiving a higher number of medications demonstrated a significantly greater occurrence of pDDIs, highlighting the strong relationship between polypharmacy and interaction risk. As the number of concurrently administered medications increases, the likelihood of pharmacokinetic and pharmacodynamic interaction pathways rises substantially. This finding aligns with previous studies that have identified polypharmacy as one of the most important determinants of drug–drug interactions in hospitalized patients [36, 37, 38].

In addition, patients with multiple comorbidities were observed to have a higher burden of pDDIs, reflecting the complexity of therapeutic regimens required to manage coexisting conditions. These findings emphasize the importance of targeted monitoring and intervention strategies in high‐risk patient groups, particularly patients exposed to polypharmacy.

Overall, the observed distribution patterns highlight the need for individualized medication management approaches and reinforce the role of clinical pharmacists in identifying and mitigating drug–drug interaction risks in hospitalized cardiovascular patients.

### 4.6 Clinical and Policy Implications

The findings of this study underscore the importance of implementing systematic and proactive approaches to identify and manage potential drug–drug interactions (pDDIs) in hospital settings. The high prevalence of pDDIs observed highlights the need to integrate drug interaction screening tools into electronic prescribing and clinical decision support systems, which can facilitate early detection of clinically significant interactions and support safer prescribing practices [39, 40, 41, 42].

In addition, strengthening interdisciplinary collaboration among physicians, pharmacists, and nurses is essential for optimizing medication safety (Berger et al., 2025; Toledo, 2025). Routine medication review and reconciliation processes, particularly during hospital admission and discharge, play a critical role in identifying and mitigating potential drug–drug interactions. Pharmacist‐led interventions have been shown to reduce medication‐related risks, improve adherence to treatment guidelines, and enhance overall patient safety outcomes [24, 43, 44].

From a policy perspective, these findings support the need for institutional and national strategies aimed at strengthening pharmacovigilance systems, particularly in resource‐limited settings. The implementation of standardized protocols for DDI screening, combined with continuous professional training and capacity building for healthcare providers, may contribute to reducing medication‐related harm and improving the quality of care for patients with cardiovascular diseases.

### 4.7 Limitations of the Study

This study has several limitations that should be considered when interpreting the findings. First, the retrospective design relied on data extracted from medical records, which may have been incomplete or subject to documentation bias, potentially leading to underestimation or misclassification of some variables. Second, the study focused on potential drug–drug interactions identified through screening tools rather than clinically confirmed adverse drug reactions. As a result, the findings do not establish direct causal relationships between identified interactions and patient outcomes, but rather highlight potential risks that may require clinical attention. Third, the sample size actually achieved (n = 100) was substantially smaller than the calculated target of 384, resulting in a wider confidence interval around the prevalence estimate (95% CI: 83.8–95.2%) than originally intended, and may have limited the statistical power to detect significant associations between certain patient characteristics, such as age and sex, and the occurrence of pDDIs. Fourth, although a systematic sampling interval was applied, the first eligible record was not selected using a randomized starting point, which may have introduced a degree of selection bias. Fifth, the structured abstraction tool did not capture renal function (creatinine or estimated glomerular filtration rate), length of hospital stay, admitting ward or service, or body mass index, as these were not included among the variables specified a priori for this medication‐focused pDDI screening study; their absence limits the ability to assess renal‐function‐dependent interaction risk (e.g., for the Diclofenac + Enalapril combination) and other clinically relevant covariates identified in comparable studies. Similarly, although residential area (Nairobi *vs.* outside Nairobi) was recorded (Table 1), it was not carried forward into the association analyses because it was not among the study's primary variables of interest; future studies should consider incorporating these clinical and geographic covariates. Additionally, sex was recorded retrospectively from clinical documentation as a binary male/female variable, which may not fully capture gender identity.

In addition, the high prevalence of pDDIs observed in this study, with a limited number of patients without interactions, restricted the use of multivariable regression models due to concerns regarding statistical stability and reliability of estimates. Consequently, the analysis was limited to chi‐square and stratified descriptive approaches. Finally, the study was conducted in a single national referral hospital, which may limit the generalizability of the findings to other healthcare settings. Future studies incorporating larger sample sizes, multicenter designs, prospective data collection, and advanced analytical methods are recommended to provide a more comprehensive understanding of drug–drug interactions and their clinical implications.

## 5 Conclusion

This study demonstrates a high prevalence of potential drug–drug interactions (pDDIs) among hospitalized patients with cardiovascular diseases, highlighting the substantial burden of medication‐related risks in this population. The predominance of moderate interactions, alongside the presence of clinically important severe interactions, underscores the need for routine screening and careful monitoring to optimize pharmacotherapy and enhance patient safety.

The frequent involvement of commonly prescribed cardiovascular drug classes—including antiplatelets, anticoagulants, antihypertensive agents, nonsteroidal anti‐inflammatory drugs (NSAIDs), and diuretics—reflects the complexity of treatment regimens and the challenges associated with polypharmacy. These findings emphasize the importance of individualized prescribing practices and vigilant medication review, particularly among patients receiving multiple medications.

From a clinical and systems perspective, the integration of drug interaction screening tools into prescribing workflows, together with strengthened interdisciplinary collaboration and pharmacist‐led medication review, may improve the detection and management of pDDIs. These approaches are especially relevant in resource‐limited settings, where structured pharmacovigilance systems are still evolving.

While this study provides important insights into the prevalence and patterns of pDDIs in a tertiary care setting, further research is warranted to evaluate the clinical outcomes associated with these interactions and to assess the effectiveness of targeted interventions, including clinical decision support systems and pharmacist‐led care models.

## Author Contributions

**Brenda Koech:** conceptualization, writing – original draft, methodology, writing – review and editing, validation, investigation. **Ermias Terefe:** conceptualization, funding acquisition, writing – original draft, writing – review and editing, methodology, project administration, supervision, resources.

## Funding

The authors have nothing to report.

## Ethics Statement

Ethical approval for this study was obtained from the United States International University–Africa Institutional Review Board (USIU‐A IRB; Approval No. USIU‐A/IRB/SS03/2023) and the National Commission for Science, Technology and Innovation (NACOSTI; Permit No. NACOSTI/P/23/23349). As this study involved retrospective review of anonymised medical records and did not involve direct patient contact or intervention, a waiver of individual informed consent was granted by the ethics committees and permission to access patient medical records was also granted by the Kenyatta National Hospital Ethics and Research Committee.

## Conflicts of Interest

The authors declare no conflicts of interest.

## Transparency Statement

The lead author and corresponding author, Dr. Ermias Mergia Terefe, affirms that this manuscript is an honest, accurate, and transparent account of the study being reported; that no important aspects of the study have been omitted; and that any discrepancies from the study as planned have been explained.

## Supporting information


Supporting File 1

Supporting File 2

## Data Availability

The data supporting the findings of this study are available from the corresponding author upon reasonable request. The data are not publicly available because they contain information derived from patient medical records obtained from Kenyatta National Hospital and are subject to institutional and ethical restrictions designed to protect patient confidentiality. Researchers who meet the criteria for access to confidential data may request the dataset from the corresponding author.
