# Supplementary material for: Assessment of Potential Drug‐Drug Interactions in Hospitalized Patients With Cardiovascular Diseases at Kenyatta National Hospital, Nairobi, Kenya: A Cross‐Sectional Study
Source: Health Sci Rep. 2026 Oct 8;9(10):e73356. doi: 10.1002/hsr2.73356 (PMC13650412; doi:10.1002/hsr2.73356)
Supplement: Supplementary file 1 [file HSR2-9-e73356-s001.docx]

**STROBE Statement — Checklist of Items That Should Be Included in Reports of Cross-Sectional Studies**

**Manuscript:** Assessment of Potential Drug–Drug Interactions in Hospitalized Patients with Cardiovascular Diseases at Kenyatta National Hospital, Nairobi, Kenya: A Cross-Sectional Study

| Item No. | Recommendation | Reported in manuscript section | Notes |
| --- | --- | --- | --- |
| 1a | Indicate the study’s design in the title or abstract | Title (“…A Cross-Sectional Study”); Abstract, Methods | Reported |
| 1b | Provide an informative and balanced summary of what was done and found | Abstract | Reported |
| 2 | Background/rationale for the investigation | Introduction | Reported |
| 3 | State specific objectives, including any prespecified hypotheses | Introduction (closing paragraph) | Reported |
| 4 | Present key elements of study design early in the paper | Methods, “Study Design” | Reported |
| 5 | Describe the setting, locations, and relevant dates | Methods, “Study Setting and Period” | Reported |
| 6a | Give eligibility criteria and the sources and methods of selection of participants | Methods, “Target Population,” “Eligibility Criteria,” “Sampling and Sample Size Determination” | Reported |
| 7 | Define all outcomes, exposures, predictors, potential confounders, and effect modifiers | Methods, “Study Variables” | Reported |
| 8 | For each variable of interest, give sources of data and details of methods of assessment | Methods, “Data Collection Methods” | Reported |
| 9 | Describe efforts to address potential sources of bias | Discussion, “Limitations” (Section 4.7) | Partially reported — selection bias from the non-randomised sampling start point and documentation bias are discussed |
| 10 | Explain how the study size was arrived at | Methods, “Sampling and Sample Size Determination”; Discussion, “Limitations” | Reported — the calculated target (n = 384) and achieved sample (n = 100) are both stated, with the shortfall discussed as a limitation |
| 11 | Explain how quantitative variables were handled; describe grouping and why | Methods, “Study Variables”; Results, Section 3.5 | Reported — age dichotomised at 50 years (a priori clinical threshold, now justified in Section 3.5) and medication count grouped into three ordinal categories |
| 12a | Describe all statistical methods | Methods, “Statistical Analysis” | Reported |
| 12b | Describe methods used to examine subgroups and interactions | Results, Section 3.5; Tables 6–8 | Reported |
| 12c | Explain how missing data were addressed | — | Not separately reported. |
| 12d | If applicable, describe sampling strategy | Methods, “Sampling and Sample Size Determination” | Reported |
| 12e | Describe any sensitivity analyses | — | Not applicable — no sensitivity analyses were conducted |
| 13a | Report numbers of individuals at each stage of the study | Methods (final sample, n = 100); Results, Section 3.1 | reported |
| 13b | Give reasons for non-participation at each stage | — | Not applicable |
| 13c | Consider use of a flow diagram | — | Not provided, |
| 14a | Give characteristics of study participants and information on exposures/confounders | Results, Section 3.1; Tables 1–2 | Reported |
| 14b | Indicate the number of participants with missing data for each variable of interest | — | Not separately reported (see Item 12c) |
| 14c | Summarise follow-up time | — | Not applicable — cross-sectional design |
| 15 | Report numbers of outcome events or summary measures | Results, Section 3.2; Table 3 | Reported |
| 16a | Give unadjusted estimates and, where applicable, confounder-adjusted estimates and their precision | Results, Sections 3.2–3.5; Tables 3, 4, 6, 8 | Reported for unadjusted associations, with 95% confidence intervals now included for prevalence and severity estimates. No multivariable/confounder-adjusted model was fitted; this is stated as a limitation (Section 4.7) |
| 16b | Report category boundaries when continuous variables were categorised | Results, Section 3.5; Tables 6, 8 | Reported (age ≤50 vs >50 years; medication count 2–4, 5–7, ≥8) |
| 16c | Translate relative risk into absolute risk for a meaningful time period, if relevant | — | Not applicable |
| 17 | Report other analyses performed | Results, Section 3.5 | Reported — Cochran–Armitage trend test presented as an additional analysis |
| 18 | Summarise key results with reference to study objectives | Discussion, Section 4.1 | Reported |
| 19 | Discuss limitations, sources of potential bias/imprecision, and direction/magnitude of bias | Discussion, Section 4.7 | Reported |
| 20 | Give a cautious overall interpretation considering objectives, limitations, and other evidence | Discussion, Sections 4.1, 4.3–4.6 | Reported |
| 21 | Discuss generalisability of the study results | Discussion, Section 4.7 | Reported |
| 22 | Give the source of funding and the role of funders | Funding Statement | Reported |
