# Supplementary material for: Assessment of Potential Drug‐Drug Interactions in Hospitalized Patients With Cardiovascular Diseases at Kenyatta National Hospital, Nairobi, Kenya: A Cross‐Sectional Study
Source: Health Sci Rep. 2026 Oct 8;9(10):e73356. doi: 10.1002/hsr2.73356 (PMC13650412; doi:10.1002/hsr2.73356)
Supplement: Supplementary file 2 [file HSR2-9-e73356-s002.docx]

**Supplementary Table S1. Complete list of identified potential drug-drug interactions among hospitalized cardiovascular patients.**

| **Drug Combination** | **Mechanism** | **Severity** | **Recommended Action** | **Frequency (n)** |
| --- | --- | --- | --- | --- |
| Phenytoin + Nifedipine | Cytochrome P450 3A4 induction resulting in reduced nifedipine levels | Severe | Avoid or use alternative | 12 |
| Diclofenac + Enalapril | Pharmacodynamic antagonism resulting in reduced antihypertensive effect and impaired renal function | Severe | Avoid or use alternative | 18 |
| Esomeprazole + Clopidogrel | Cytochrome P450 2C19 inhibition resulting in reduced clopidogrel activation | Severe | Avoid or use alternative | 22 |
| Phenytoin + Losartan | Cytochrome P450 3A4 induction resulting in reduced losartan levels | Severe | Avoid or use alternative | 10 |
| Aspirin + Enalapril | Prostaglandin inhibition may attenuate the antihypertensive effect of enalapril and increase renal risk in susceptible patients | Moderate | Monitor blood pressure and renal function; use the lowest effective aspirin dose | 20 |
| Esomeprazole + Digoxin | Increased gastric pH resulting in increased digoxin levels | Severe | Avoid or use alternative | 8 |
| Efavirenz + Carvedilol | Potential CYP2C9 inhibition and induction of glucuronidation; net effect on carvedilol exposure is uncertain | Potential weak interaction | No a priori dose adjustment; monitor clinically | 5 |
| Levofloxacin + Ondansetron | Increased risk of QT interval prolongation | Severe | Avoid or use alternative | 14 |
| Ceftriaxone + Enoxaparin | Increased bleeding risk | Severe | Avoid or use alternative | 9 |
| Nifedipine + Amlodipine | Potential pharmacokinetic interaction and additive vasodilatory effects | Severe | Avoid or use alternative | 28 |
| Omeprazole + Phenytoin | Cytochrome P450 2C9 inhibition resulting in increased phenytoin levels | Moderate | Monitor closely | 11 |
| Phenytoin + Ondansetron | Cytochrome P450 3A4 induction resulting in reduced ondansetron exposure | Moderate | Monitor closely | 10 |
| Metronidazole + Phenytoin | Cytochrome P450 2C9 inhibition resulting in increased phenytoin levels | Moderate | Monitor closely | 9 |
| Omeprazole + Losartan | Reduced conversion to active metabolite | Moderate | Monitor closely | 7 |
| Aspirin + Losartan | Pharmacodynamic antagonism | Moderate | Monitor closely | 18 |
| Aspirin + Enoxaparin | Additive anticoagulant effect | Moderate | Monitor closely | 22 |
| Enoxaparin + Clopidogrel | Increased bleeding risk | Moderate | Monitor closely | 25 |
| Enalapril + Spironolactone | Increased risk of hyperkalemia | Moderate | Monitor closely | 15 |
| Enalapril + Furosemide | Increased risk of hypotension and renal impairment | Moderate | Monitor closely | 20 |
| Spironolactone + Diclofenac | Increased risk of hyperkalemia | Moderate | Monitor closely | 14 |
| Aspirin + Warfarin | Increased anticoagulant effect and bleeding risk | Moderate | Monitor closely | 20 |
| Aspirin + Clopidogrel | Increased bleeding risk | Moderate | Monitor closely | 29 |
| Clopidogrel + Warfarin | Increased anticoagulant effect and bleeding risk | Moderate | Monitor closely | 18 |
| Azithromycin + Atorvastatin | Cytochrome P450 3A4 inhibition | Moderate | Monitor closely | 14 |
| Labetalol + Amlodipine | Additive hypotensive effects | Moderate | Monitor closely | 28 |
